# Overlooked Binary Compounds Uncovered in the Reinspection of the La–Au System: Synthesis, Crystal Structures, and Electronic Properties of La_7_Au_3_, La_3_Au_2_, and La_3_Au_4_

**DOI:** 10.1021/acs.inorgchem.1c01355

**Published:** 2021-07-28

**Authors:** Alexander Ovchinnikov, Anja-Verena Mudring

**Affiliations:** Department of Materials and Environmental Chemistry, Stockholm University, Svante Arrhenius väg 16 C, 10691 Stockholm, Sweden

## Abstract

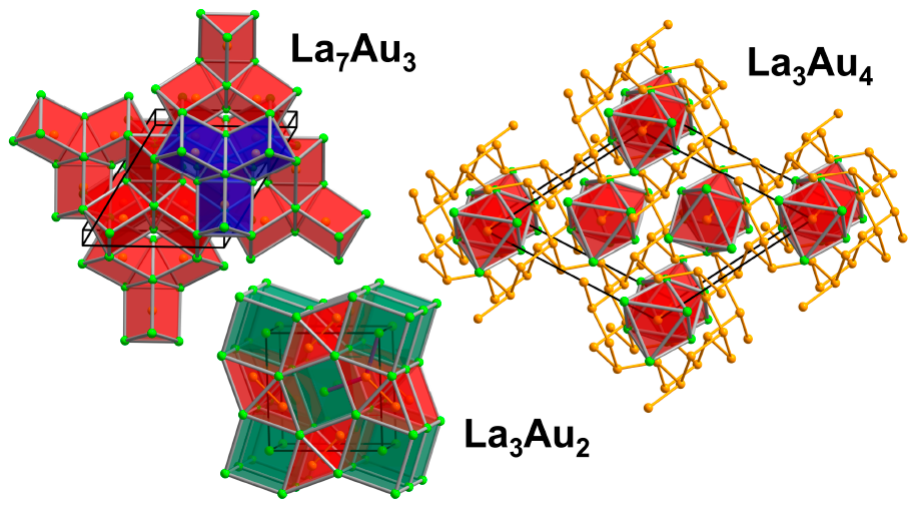

Although compound formation between two elements is well studied, thorough investigations make it possible to uncover new binary compounds. A re-examination of the La–Au system revealed three new phases, which were characterized with respect to their structural and electronic properties as well as thermal stability: La_7_Au_3_ (Th_7_Fe_3_ type, space group *P*6_3_*mc*, Pearson code *hP*20) appears to be metastable. It can be obtained by slow crystallization from a stoichiometric melt. La_3_Au_2_ (U_3_Si_2_ type, space group *P*4/*mbm*, Pearson code *tP*10) is stable up to 1013 K, where it decomposes peritectically. La_3_Au_4_ (Pu_3_Pd_4_ type, space group *R*3̅, Pearson code *hR*14) is thermally stable up to at least 1273 K. In addition, the crystal structures of La_2_Au (*anti*-PbCl_2_ type, space group *Pnma*, Pearson code *oP*12) and α-LaAu (FeB type, space group *Pnma*, Pearson code *oP*8) could be determined by single-crystal X-ray diffraction. The electronic structures and chemical bonding have been evaluated from first principles calculations. They show that all compounds can be viewed as electron-rich, polar intermetallics.

## Abstract 2

Revisiting the La−Au phase diagram unveils three new binary phases

## Introduction

The location of gold in the sixth period of the periodic table lends this element physical and chemical characteristics that are often strikingly different from those of the lighter congeners in group 11.^1^ To a large extent, these differences are associated with strong relativistic effects impacting the electronic properties of the gold valence states.^1,2^ In particular, relativistic stabilization of the 6s^2^ electronic configuration results in a high electronegativity of gold, comparable to that of iodine on the Pauling scale,^3^ although some recently developed electronegativity scales suggest a somewhat lower value for Au in comparison to I.^4,5^ For this reason, compounds of Au with other metals frequently reveal a significant transfer of the electron density to the Au atoms, which allows their description as aurides, i.e., phases with anionic Au species. Arguably, the most illustrative and well-studied example is the ionic compound (Cs^+^)(Au^–^), adopting the CsCl structure type and displaying an optical band gap of 2.5 eV.^6−8^ The highly ionic nature of this salt-like material is also reflected in its ability to form crystal solvates, such as CsAu·NH_3_.^9^ Quite often, gold and iodine compounds are found to be isostructural, e.g., CsAu and CsI and oxygen-containing phases with anionic Au or I: A_3_XO (A = K, Rb, Cs; X = I, Au)^10^ and (AX)_2_(A_3_AuO_2_) (A = K, Rb; X = I, Au).^11,12^ Similar to iodine, anionic gold forms polyanions, albeit they rarely are isotypic. The Au substructures in aurides form polyanions of various dimensionalities, ranging from isolated Au atoms to three-dimensional frameworks.^1^ This opens up possibilities for crystal structure design, which likely have not been brought to full potential yet.

From the physics perspective, the relativistic properties of gold, for instance, strong spin–orbit coupling, may be utilized to induce and tune various physical phenomena, especially in multinary Au-containing systems, where higher structural flexibility can be achieved. Examples include topologically nontrivial electronic states^13^ and unconventional magnetic orders and spin dynamics in compounds of Au with magnetic elements.^14^

Gold-containing intermetallics are conventionally produced by annealing Au with other metals at elevated temperatures, usually close to or above the melting point of some or all of the reactants. In many cases, induction or arc furnaces are utilized for these purposes.^15−18^ Because of the high reaction temperatures, mainly thermodynamically stable compounds are produced by such methods. Since diffusion rates drop considerably with decreasing temperature, targeting metastable or low-temperature phases is challenging, especially when various compositionally close phases exist in the system.^19^ Long annealing times are necessary to afford the completion of solid-state reactions at low temperatures. Alternatively, chemical activities can be increased by utilizing suitable media, e.g., low-melting metal fluxes or molten salts. Although the flux approach has yielded many intermetallic compounds not easily accessible by conventional high-temperature methods,^20−24^ its application to Au-containing compositions remains rather limited,^25−27^ mostly because the intended reaction partner for gold often forms stable phases with the flux constituents instead.

In this contribution, we present the synthesis and characterization of three new binary La aurides—La_7_Au_3_, La_3_Au_2_, and La_3_Au_4_—which were discovered by us first during an investigation of the ternary systems La–TM–Au, where TM is a magnetic transition metal. Our initial motivation was to design materials with unusual magnetic arrangements, such as noncollinear magnetic structures. The titular binary phases came out as a side result of those exploratory efforts. Subsequently, we targeted these compounds by re-examining the binary La–Au system. While La_7_Au_3_ appears to be metastable, La_3_Au_2_ and La_3_Au_4_ are thermodynamically stable phases, although La_3_Au_2_ exhibits a rather limited thermal stability window. In addition, we re-examined the compositionally close compounds La_2_Au and LaAu. Electronic structure calculations and bonding analysis reveal that all studied materials are metallic and belong to the class of polar intermetallics and hence can be described as aurides.

## Experimental Section

### Synthesis

All weighing and mixing procedures were carried out in an Ar-filled glovebox with a controlled atmosphere. Single-phase polycrystalline samples of La_3_Au_2_ and La_3_Au_4_ were prepared by a two-step procedure. First, stoichiometric mixtures of La (HEFA Rare Earth, 99.9 wt %) and Au (NEYCO, 99.999 wt %) were arc-melted three times to ensure homogeneity. The weight losses at this step were smaller than 0.6 wt %. The as-cast buttons were wrapped in Mo foil and sealed in evacuated fused silica tubes. The tubes were loaded in box furnaces and annealed at 973 K over a period of 10 days for La_3_Au_2_ and at 1073 K over a period of 7 days for La_3_Au_4_. To reach the target temperatures a heating rate of 200 K/h was applied. After the annealing step, the furnaces were switched off, and the samples were allowed to cool to room temperature naturally. It is worth noting that in the case of La_3_Au_4_, the sample came out single-phase directly after arc melting. Prolonged annealing helped to improve crystallinity.

The La-richest phase La_7_Au_3_ could not be prepared as single-phase. The cleanest sample containing about 30 wt % La_7_Au_3_ was produced by combining the elements with the stoichiometric ratio by arc melting and melting the as-cast pellet at 873 K in a Mo boat jacketed in an evacuated fused silica tube, followed by cooling to 773 K at a rate of 5 K/h.

To check for possible stabilization of La_7_Au_3_ by the presence of hydrogen, occasionally reported in intermetallic compounds,^28^ we attempted the remelting of an as-cast button with the nominal composition La_7_Au_3_ under hydrogenating conditions. For this purpose, the button was placed in an alumina crucible sealed in an evacuated fused silica tube along with a small amount of TiH_2_ powder (Alfa Aesar, ≥99 wt %), loaded in a separate alumina crucible and topped with quartz wool. The employed heating program was the same as the one described above for the La_7_Au_3_ sample. The reported onset of thermal decomposition of TiH_2_ is around 673 K.^29^ The used amount of TiH_2_ corresponded to a H_2_ pressure of 500 mbars at *T* = 873 K if complete decomposition of TiH_2_ into Ti metal and H_2_ is assumed. Of course, this value is an upper estimate. The hydrogenation reaction did not result in the formation of La_7_Au_3_, producing LaH_2_ and an unidentified crystalline product instead.

The crystal structures of La_2_Au and LaAu, the phases located in the compositional vicinity of the newly discovered binaries, were never accurately determined, which motivated us to conduct crystallographic studies for these compositions as well. Single crystals of the low-temperature modification α-LaAu were found in a sample with nominal composition La_3_Au_2_ annealed at 1273 K for 5 h and cooled to room temperature at a rate of 5 K/h. Another La-rich binary auride, La_2_Au, was reproducibly observed in samples during exploratory synthesis attempts. Because of their high ductility and malleability, La_2_Au single crystals prepared by high-temperature treatment of the elements were not of sufficient quality for X-ray diffraction analysis. Suitable crystals were grown using a La flux. A mixture of La and Au with the molar ratio 10:3, respectively, was loaded in a Ta tube sealed at one end. After that, the tube was welded shut under high-purity Ar using a custom-built arc welder and placed in an evacuated fused silica tube. The mixture was heated to 1223 K at a rate of 200 K/h, kept at this temperature for 5 h, and cooled to 773 K at a rate of 5 K/h. At this point, the heating was turned off and the sample was cooled to room temperature. The Ta tube was cut open inside the glovebox. Submillimeter-sized single crystals of La_2_Au were mechanically extracted from the La matrix.

For the binary compounds in this contribution, we attempted crystal growth from a Pb flux, as a more convenient alternative for long annealing in the solid state. However, in all cases, LaPb_3_ was the main product and no binary La–Au phases were produced.

### Powder X-ray Diffraction (PXRD)

PXRD patterns were collected in reflection geometry on a PANalytical X’Pert diffractometer (Cu Kα1 λ = 1.54056 Å) and on a Bruker Phaser D2 diffractometer (filtered Cu Kα λ_mean_ = 1.5418 Å) in the 2θ range of 5–90° with a step size of 0.013°. The samples were immobilized on low-background silicon holders with vacuum grease. Rietveld refinements were carried out using the JANA2006 software.^30^

### Single-Crystal X-ray Diffraction (SCXRD)

Suitable crystals were selected under vacuum grease and mounted on low-background plastic loops. Data collection for all studied compounds was performed at room temperature on a Bruker D8 Venture (Mo Kα λ = 0.71073 Å) equipped with a PHOTON 100 CMOS detector. In addition, a low-temperature measurement (*T* = 100 K) was conducted for a crystal of La_3_Au_4_. For this purpose, the crystal was cooled with a stream of nitrogen using an OxfordCryosystems cooling setup. Data integration and absorption corrections were performed using the SAINT^31^ and SADABS^32^ software, respectively. Crystal structures were solved by dual-space methods as implemented in SHELXT^33^ and refined by a full-matrix least-squares method on *F*^2^ with SHELXL.^34^ Atomic coordinates were standardized using STRUCTURE TIDY.^35^ Details of the data collection and selected crystallographic parameters are summarized in Tables 1–12 (room-temperature measurements) and S1–S3 (low-temperature measurements).

**Table 1:** Refinement Details and Selected Crystallographic Data for La_7_Au_3_, La_3_Au_2_, and La_3_Au_4_ (Room Temperature, Mo Kα, λ = 0.71073 Å)

refined composition	La_7_Au_3_	La_3_Au_2_	La_3_Au_4_
CCDC number	2072067	2072068	2072069
fw/g mol^–1^	1563.27	810.66	1204.60
space group	*P*6_3_*mc* (no. 186)	*P*4/*mbm* (no. 127)	*R*3̅ (no. 148)
*Z*	2	2	6
*a*/Å	10.5726(7)	8.431(3)	14.038(3)
*c*/Å	6.5801(5)	4.0618(15)	6.2248(5)
*V*/Å^3^	636.98(10)	288.7(2)	1062.4(5)
ρ_calc_/g cm^–3^	8.151	9.325	11.297
μ_Mo Kα_/mm^–1^	57.24	72.18	100.08
*R*_int_	0.042	0.046	0.055
*R*_1_ [*I* > 2σ(*I*)]^a^	0.022	0.024	0.022
*wR*_2_ [*I* > 2σ(*I*)]^a^	0.038	0.053	0.047
*R*_1_ [all data]^a^	0.026	0.026	0.026
*wR*_2_ [all data]^a^	0.038	0.053	0.048
Flack parameter	0.029(9)		
Δρ_max,min_/e Å^–3^	1.36, −1.16	1.35, −1.38	1.72, −1.15

a *R*_1_ = ∑||*F*_0_| – |*F*_c_||/∑|*F*_0_|; *wR*_2_ = [∑[*w*(*F*_0_^2^ – *F*_c_^2^)^2^]/∑[*w*(*F*_0_^2^)^2^]]^1/2^, where *w* = 1/[σ^2^*F*_0_^2^ + (*AP*)^2^ + (*BP*)] and *P* = (*F*_0_^2^ + 2*F*_c_^2^)/3. *A* and *B* are the respective weight coefficients. (See the CIF data.)

**Table 2:** Refinement Details and Selected Crystallographic Data for La_2_Au and α-LaAu (Room Temperature, Mo Kα λ = 0.71073 Å)

refined composition	La_2_Au	LaAu
CCDC number	2072070	2072071
fw/g mol^–1^	474.79	335.88
space group	*Pnma* (no. 62)	*Pnma* (no. 62)
*Z*	4	4
*a*/Å	7.5064(12)	7.6053(11)
*b*/Å	5.1531(8)	4.8235(8)
*c*/Å	9.5036(13)	5.9234(8)
*V*/Å^3^	367.61(10)	217.29(6)
ρ_calc_/g cm^–3^	8.579	10.267
μ_Mo Kα_/mm^–1^	62.35	86.33
*R*_int_	0.038	0.037
*R*_1_ [*I* > 2σ(*I*)]^a^	0.042	0.025
*wR*_2_ [*I* > 2σ(*I*)]^a^	0.106	0.059
*R*_1_ [all data]^a^	0.047	0.026
*wR*_2_ [all data]^a^	0.109	0.059
Δρ_max,min_/e Å^–3^	3.50, −2.49	1.74, −1.59

a *R*_1_ = ∑||*F*_0_| – |*F*_c_||/∑|*F*_0_|; *wR*_2_ = [∑[*w*(*F*_0_^2^ – *F*_c_^2^)^2^]/∑[*w*(*F*_0_^2^)^2^]]^1/2^, where *w* = 1/[σ^2^*F*_0_^2^ + (*AP*)^2^ + (*BP*)] and *P* = (*F*_0_^2^ + 2*F*_c_^2^)/3. *A* and *B* are the respective weight coefficients (see the CIF data).

**Table 3:** Atomic Coordinates and Equivalent Isotropic Displacement Parameters (Å^2^) for La_7_Au_3_

atom	site	*x*	*y*	*z*	*U*_eq_^a^
La1	6*c*	0.53800(5)	1 – *x*	0.0245(3)	0.0220(2)
La2	6*c*	0.87362(5)	1 – *x*	0.3121(3)	0.0214(2)
La3	2*b*	1/3	2/3	0^b^	0.0217(3)
Au	6*c*	0.19023(3)	1 – *x*	0.2519(3)	0.02214(15)

a *U*_eq_ is defined as one-third of the trace of the orthogonalized *U_ij_* tensor.

b The *z* coordinate of La3 was fixed to 0 after data standardization.

**Table 4:** Atomic Coordinates and Equivalent Isotropic Displacement Parameters (Å^2^) for La_3_Au_2_

atom	site	*x*	*y*	*z*	*U*_eq_^a^
La1	4*h*	0.16362(8)	*x* + 1/2	1/2	0.0214(3)
La2	2*a*	0	0	0	0.0284(4)
Au	4*g*	0.62723(5)	*x* – 1/2	0	0.0194(2)

a *U*_eq_ is defined as one-third of the trace of the orthogonalized *U_ij_* tensor.

**Table 5:** Atomic Coordinates and Equivalent Isotropic Displacement Parameters (Å^2^) for La_3_Au_4_

atom	site	*x*	*y*	*z*	*U*_eq_^a^
La	18*f*	0.04359(5)	0.21220(5)	0.23186(8)	0.02253(18)
Au1	18*f*	0.39091(3)	0.11467(3)	0.04913(6)	0.02213(15)
Au2	3*b*	0	0	1/2	0.0370(3)
Au3	3*a*	0	0	0	0.0426(3)

a *U*_eq_ is defined as one-third of the trace of the orthogonalized *U*_*ij*_ tensor.

**Table 6:** Atomic Coordinates and Equivalent Isotropic Displacement Parameters (Å^2^) for La_2_Au

atom	site	*x*	*y*	*z*	*U*_eq_^a^
La1	4*c*	0.00819(18)	1/4	0.67556(14)	0.0244(4)
La2	4*c*	0.1515(2)	1/4	0.08642(13)	0.0260(4)
Au	4*c*	0.24308(13)	1/4	0.40218(9)	0.0278(3)

a *U*_eq_ is defined as one-third of the trace of the orthogonalized *U*_*ij*_ tensor.

**Table 7:** Atomic Coordinates and Equivalent Isotropic Displacement Parameters (Å^2^) for α-LaAu

atom	site	*x*	*y*	*z*	*U*_eq_^a^
La	4*c*	0.18451 (11)	1/4	0.64246(14)	0.0174(3)
Au	4*c*	0.03896 (8)	1/4	0.14532(9)	0.0188(3)

a *U*_eq_ is defined as one-third of the trace of the orthogonalized *U*_*ij*_ tensor.

**Table 8:** Selected Interatomic Distances (Å) in La_7_Au_3_

atoms		distance
La1	–Au × 2	3.0761(8)
	–La1 × 2	3.5722(7)
	–La3	3.7513(9)
	–La2 × 2	3.8164(9)
	–La3	3.9166(16)
	–La2 × 2	4.0197(7)
	–La1 × 2	4.0812(15)
	–La3	4.1791(16)
La2	–Au × 2	3.0962(5)
	–Au	3.1213(12)
	–La1 × 2	3.8164(8)
	–La3	3.9863(10)
	–La2 × 2	4.0086(15)
	–La1 × 2	4.0198(7)
	–La2 × 4	4.0225(5)
La3	–Au × 3	3.1008(10)
	–La1 × 3	3.7513(8)
	–La1 × 3	3.9167(16)
	–La2 × 3	3.9862(10)
	–La1 × 3	4.1791(16)
Au	–La1 × 2	3.0760(8)
	–La2 × 2	3.0962(4)
	–La3	3.1008(10)
	–La2	3.1213(12)

**Table 9:** Selected Interatomic Distances (Å) in La_3_Au_2_

atoms		distance
La1	–Au × 4	3.1987(8)
	–Au × 2	3.2161(11)
	–La2 × 4	3.7510(8)
	–La1	3.902(2)
	–La1 × 2	4.0618(15)
La2	–Au × 4	3.3208(3)
	–La1 × 8	3.7510(8)
	–La2 × 2	4.0618(15)
Au	–Au	3.0339(13)
	–La1 × 4	3.1987(8)
	–La1 × 2	3.2161(11)
	–La2 × 2	3.3208(3)

**Table 10:** Selected Interatomic Distances (Å) in La_3_Au_4_

atoms		distance
La	–Au1	3.0059(8)
	–Au3	3.0836(6)
	–Au1	3.1205(9)
	–Au2	3.1955(6)
	–Au1	3.2225(10)
	–Au1	3.2313(10)
	–Au1	3.3140(10)
	–Au1	3.3505(7)
	–Au1	3.4390(8)
	–La	3.6540(12)
	–La × 2	3.9696(10)
	–La × 2	4.1503(9)
	–La × 2	4.3092(11)
Au1	–La	3.0058(8)
	–Au1	3.0407(8)
	–La	3.1205(9)
	–Au1 × 2	3.1836(6)
	–La	3.2226(7)
	–La	3.2312(10)
	–La	3.3140(10)
	–La	3.3504(7)
	–La	3.4390(9)
Au2	–Au3 × 2	3.1124(8)
	–La × 6	3.1955(7)
Au3	–La × 6	3.0835(6)
	–Au2 × 2	3.1124(8)

**Table 11:** Selected Interatomic Distances (Å) in La_2_Au

atoms		distance
La1	–Au	3.1399(17)
	–Au × 2	3.2775(10)
	–La2 × 2	3.6321(14)
	–La2	3.656(2)
	–La2 × 2	3.7257(15)
	–La1 × 2	4.0110(11)
	–La2	4.0502(19)
	–La1 × 2	4.218(2)
La2	–Au	3.0678(18)
	–Au	3.0785(16)
	–Au × 2	3.2141(10)
	–La1 × 2	3.6322(14)
	–La1	3.656(2)
	–La1 × 2	3.7257(15)
	–La2 × 2	3.809(2)
	–La1	4.0502(19)
Au	–La2	3.0678(18)
	–La2	3.0785(16)
	–La1	3.1399(17)
	–La2 × 2	3.2141(10)
	–La1 × 2	3.2775(10)

**Table 12:** Selected Interatomic Distances (Å) in α-LaAu

atoms		distance
La	–Au	3.1459(10)
	–Au	3.1777(10)
	–Au	3.1894(11)
	–Au × 2	3.2000(7)
	–Au × 2	3.2070(7)
	–La × 4	3.9472(6)
	–La × 2	4.0104(7)
	–La × 2	4.0671(14)
Au	–Au × 2	3.0218(7)
	–La	3.1459(10)
	–La	3.1777(10)
	–La	3.1894(11)
	–La × 2	3.2000(7)
	–La × 2	3.2070(7)

### First-Principles Calculations

Total energy calculations were performed for the newly discovered phases and some known compounds in the La–Au binary system using the Vienna ab initio Simulation Package (VASP).^36^ Exchange and correlation were described in the generalized-gradient approximation (GGA) with the Perdew–Burke–Ernzerhof exchange-correlation functional (PBE).^37^ The plane wave energy cutoff was set to 500 eV. *k*-point grids with 0.1 Å^–1^ spacing were used to sample the Brillouin zones. The structures were fully relaxed to residual forces of less than 0.02 eV/Å. All calculations were made at the scalar-relativistic level. We also checked the effect of spin–orbit coupling (SOC) on the thermodynamic properties calculated at 0 K and found that the enthalpies of formation were affected by less than 3% with SOC included. Such small differences have no effect on the conclusions drawn from the calculations made without SOC. For this reason and due to the higher computational cost of calculations with SOC, we conducted a detailed analysis of thermodynamic stability without a consideration of spin–orbit coupling. An evaluation of the phase diagrams at 0 K was performed by employing tools available within the atomic simulation environment.^38^

Electronic structure calculations were performed for La_7_Au_3_, La_3_Au_2_, and La_3_Au_4_ as well as La_2_Au and α-LaAu at the scalar relativistic level with the TB-LMTO-ASA code.^39^ The von Barth–Hedin implementation of the LDA functional was employed.^40^ For all structures except La_3_Au_2_, an introduction of empty spheres was necessary to satisfy the atomic sphere approximation (ASA). Chemical bonding was analyzed with the aid of crystal orbital Hamilton population curves,^41^ generated by the dedicated module in the LMTO program.

### Differential Scanning Calorimetry (DSC)

Differential scanning calorimetry (DSC) was performed with a computer-controlled Netzsch STA 449 F5 Jupiter thermal analyzer. Measurements were carried out at temperatures of up to 1273 K with a heating rate of 10 K/min under a flow of high-purity Ar (grade 5.0, 70 mL/min). The samples were placed in alumina pans covered with lids. Since the studied samples showed indications of side reactions with the crucible material above 1273 K, all measurements were made below this temperature.

### Magnetization Measurements

Magnetization was measured for La_3_Au_2_ and La_3_Au_4_ between 3 and 300 K in static fields from 10 to 60 000 Oe on a Quantum Design Physical Property Measurement System (PPMS) using the vibrating sample magnetometer (VSM) option. The polycrystalline samples were enclosed in polypropylene (PP) sample containers. The data were corrected for the empty holder contribution and ferromagnetic impurities using the Honda–Owen method.^42,43^

## Results and Discussion

### Synthesis

New phases La_7_Au_3_ and La_3_Au_4_ were initially identified in samples prepared during our exploratory investigations in the La–TM–Au systems, where TM is a transition element. The La_3_Au_2_ phase was discovered for the first time in a sample targeted at the preparation of pure La_7_Au_3_. For La_7_Au_3_ and La_3_Au_2_, early synthesis attempts at temperatures *T* = 1073–1273 K, i.e., close to the melting points of the elements, always resulted in the phases predicted by the published La–Au phase diagram.^44^ However, our total energy *ab initio* calculations suggested that the enthalpies of decomposition for La_7_Au_3_ and La_3_Au_2_ at *T* = 0 K must be very small (*vide infra*), which motivated us to carry out our reactions at lower temperatures.

**Figure 1:**
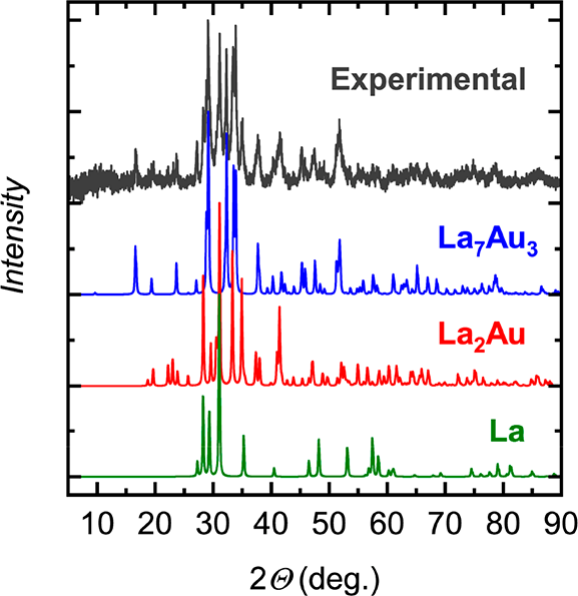
Powder X-ray diffraction pattern (Cu Kα) of a sample containing about 30 wt % La_7_Au_3_. Experimental data with subtracted background and theoretical powder patterns for La_7_Au_3_, La_2_Au, and La are shown in gray, blue, red, and green, respectively.

For La_7_Au_3_, annealing of the stoichiometric elemental mixtures or homogeneous pellets prepared by arc melting did not result in the formation of the target phase, even after heat treatment for 5 months at temperatures of as low as 673 K. This observation is in line with the results of a recent study on the La-rich side of the ternary La–Mg–Au phase diagram at 673 K, where the authors did not observe any unreported binary La–Au compounds close to the La:Au = 7:3 composition.^45^ However, we found that La_7_Au_3_ can be prepared with a reasonably high yield by slow crystallization from a melt with the same nominal composition. The samples produced this way always contained some La_2_Au and La, which are the phases expected to be in equilibrium at the given composition according to the published phase diagram.^44^ The presence of the former compound in the samples precluded accurate Rietveld refinements of the powder patterns due to the high malleability of La_2_Au and associated severe preferred orientation and anisotropic peak broadening (Figure 1). However, the amount of La_7_Au_3_ in these samples can be estimated to be around 30 wt % on the basis of the calculated corundum ratios for the phases present in the sample.^46^ Interestingly, quenching of the stoichiometric melt did not produce La_7_Au_3_, indicating that slow crystallization is essential for the formation of this phase. The failure to produce pure samples of La_7_Au_3_ is probably related to the metastability of this phase rather than stabilization by undetected foreign elements, such as hydrogen. Our attempt to prepare La_7_Au_3_ under hydrogenating conditions, as described in the Experimental Section, led to LaH_2_ and an unidentified air-sensitive crystalline product, as suggested by PXRD analysis, and no traces of La_7_Au_3_ were detected (Figure S1).

**Figure 2:**
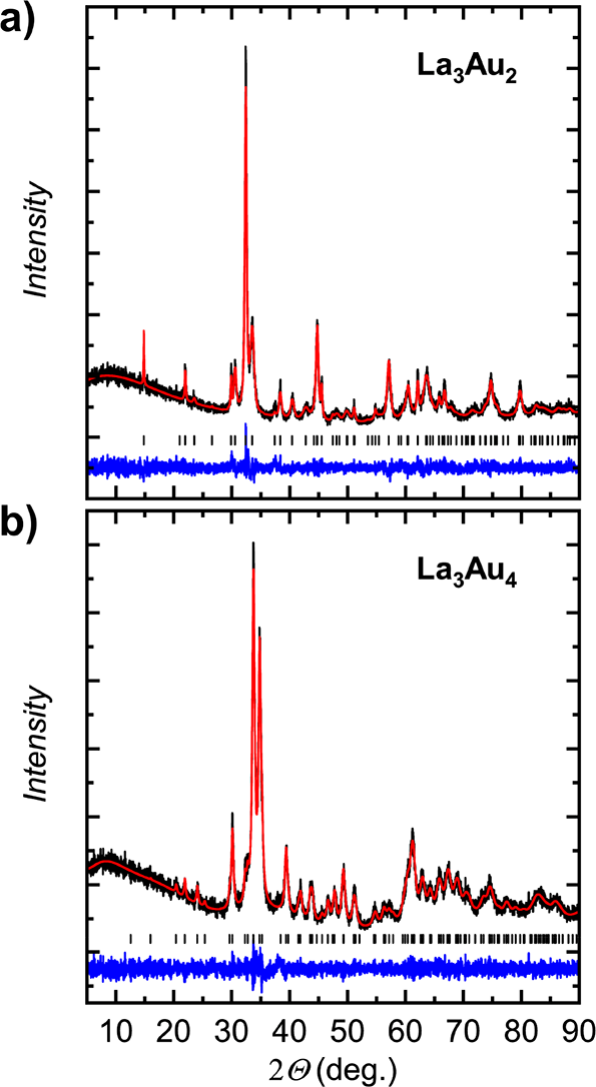
Powder X-ray diffraction patterns (Cu Kα1) and the respective Rietveld refinement for (a) La_3_Au_2_ and (b) La_3_Au_4_. The experimental data, theoretical pattern, and difference curve are shown in black, red, and blue, respectively. Tick marks correspond to the positions of the Bragg peaks.

Phase-pure samples of La_3_Au_2_ were initially obtained after annealing an as-cast pellet at 873 K for 30 days (Figure 2a). After the thermal stability of this phase had been established (*vide infra*), we managed to optimize the synthesis by increasing the annealing temperature up to 973 K, which allowed us to reduce the annealing time to 10 days.

In contrast to La_7_Au_3_ and La_3_Au_2_, La_3_Au_4_ can be easily prepared by arc melting of the elements. Prolonged annealing at temperatures of between 1073 and 1273 K improved the crystallinity without resulting in any decomposition (Figure 2b).

None of the studied phases displayed any appreciable homogeneity range, according to powder X-ray diffraction (PXRD).

### Crystal Structures

#### La_7_Au_3_

**Figure 3:**
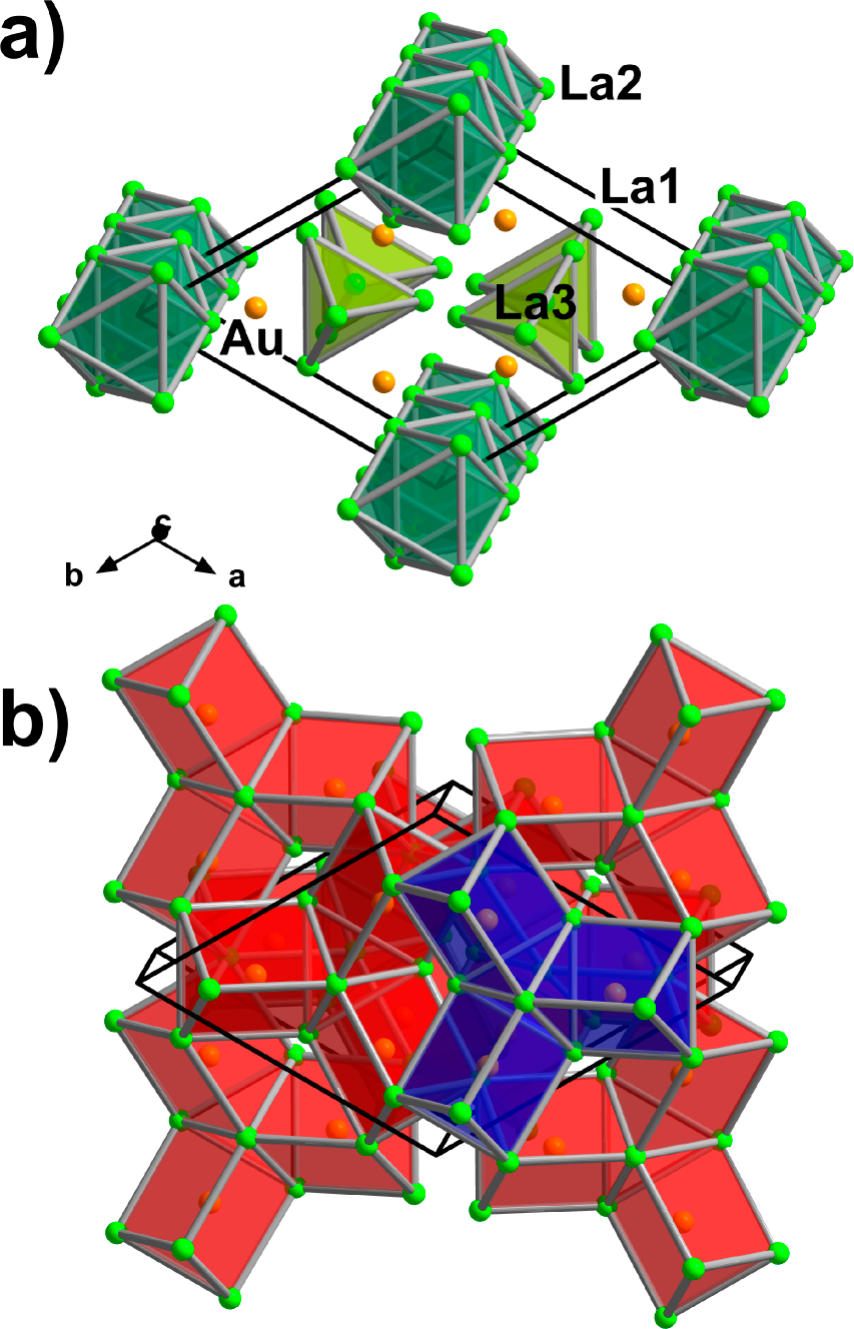
Crystal structure of La_7_Au_3_. (a) Polyhedral representation of the La substructure consisting of isolated La_4_≡(La3)(La1)_3_ tetrahedra and face-sharing [(La2)_6/2_]. (b) Interlinking of the gold-centered [AuLa_2/2_La_4/3_] trigonal prisms. A single trimer composed of three edge-shared prisms is emphasized in blue. The unit cell is outlined in black.

The La-richest phase in the La–Au system, La_7_Au_3_, crystallizes in a noncentrosymmetric structure with the Th_7_Fe_3_ type (space group *P*6_3_*mc*, Pearson code *hP*20, Figure 3). The La substructure can be viewed as consisting of isolated La_4_≡(La3)(La1)_3_ tetrahedra and columns of face-sharing [(La2)_6/2_] octahedra propagating along [001] (Figure 3a). The shortest La–La contacts in the tetrahedra and octahedra measure 4.081(2) and 4.009(2) Å, respectively. These values significantly exceed the shortest metal–metal contacts in elemental La (*d*_La–La_ = 3.74–3.77 Å^47^). The smallest La–La interatomic separation in the structure with *d*_La–La_ = 3.5722(7) Å is observed between the corners of adjacent La_4_ tetrahedra.

The unique Au site in the structure is 6-fold coordinated by La, adopting a trigonal-prismatic environment, with the Au–La distances ranging from 3.0760(8) Å to 3.121(1) Å (Figure 3b). These values are close to the numbers reported in the literature for bonding contacts in other La aurides.^48−50^ The prisms form trimers by sharing edges of the triangular bases. The trimers arrange in turn in hexagonal close packing and link by corners to build a three-dimensional structure. The shortest Au–La distance between the adjacent trimers measures 3.6512(8) Å and significantly exceeds the typical bonding contacts.

Among the gold-containing phases, the Th_7_Fe_3_ type is adopted by M_7_Au_3_ with M = Sr, Eu, and Yb,^51−53^ i.e., the metals that have a tendency to adopt the +II oxidation state. Rare earth metals (RE) that prefer the +III oxidation state form compounds with the Th_7_Fe_3_ structure when combined with transition elements of groups 8–10.^54−66^ In addition, binary compounds Sc_7_P_3_ and La_7_Ge_3_, containing main-group elements, were reported to crystallize in this type.^67,68^ From these examples, it is clear that the existence field of the Th_7_Fe_3_ structure extends over a wide region of valence electron counts. However, it is not clear at the moment if other RE_7_Au_3_ compounds exist and whether any of them are thermodynamically stable.

#### La_3_Au_2_

**Figure 4:**
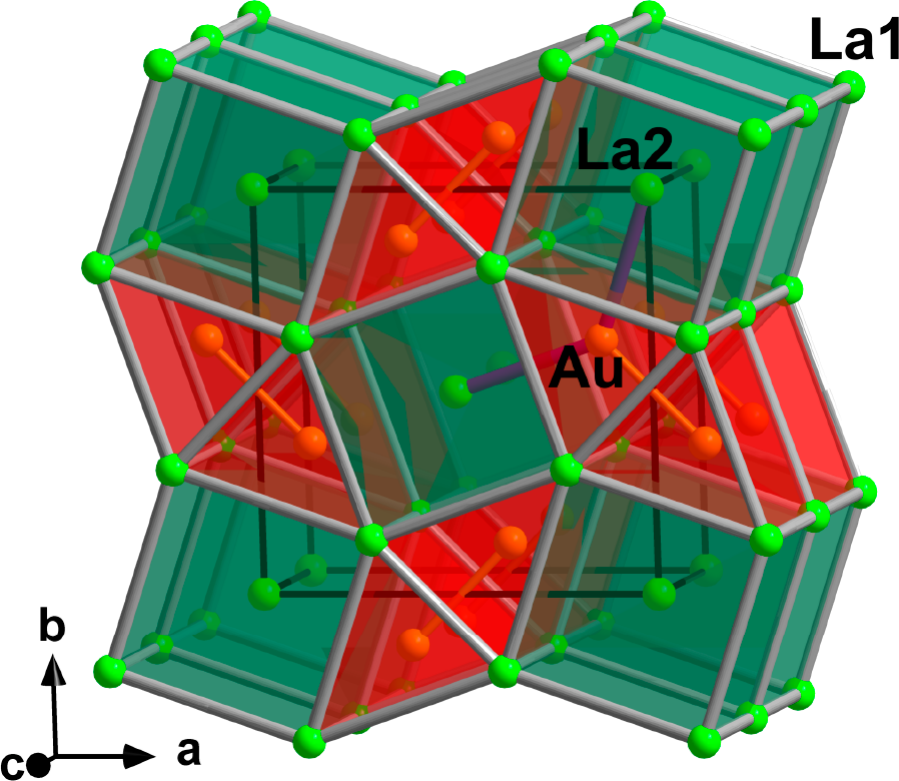
Crystal structure of La_3_Au_2_. Columns of face-sharing La-centered [(La2)(La1)_8/4_] cubes and double columns of face-sharing Au-centered [Au(La1)_6/6_] trigonal prisms are depicted in green and red, respectively. The coordination of one Au atom by La2 is shown in violet. The unit cell is outlined in black.

Binary auride La_3_Au_2_ adopts the U_3_Si_2_ structure type (space group *P*4/*mbm*, Pearson code *tP*10, Figure 4). There are two symmetrically independent La sites and a unique Au position. The La part of the structure can be visualized as consisting of La-centered [(La2)(La1)_8/4_] cubes, with *d*_La2–La1_ = 3.7510(8) Å. The cubes connect by sharing faces along the [001] direction, forming columns which interlink by edge sharing. The resulting substructure hosts extended voids propagating along the *c* direction. These voids accommodate Au_2_ dumbbells with an Au–Au distance of 3.034(1) Å. Although this distance is longer than the shortest contact in elemental Au (*d*_Au–Au_ = 2.88 Å^69^), it is in good agreement with the values reported for other structures with polyanionic Au species.^70,71^ The Au atoms are 6-fold coordinated by La1 (*d*_Au–La1_ = 3.1987(8)–3.216(1) Å), building trigonal prisms, similar to the Au coordination polyhedra in La_7_Au_3_. The prisms share faces, giving rise to extended double columns running along the *c* direction, which interlink by edge sharing. The La2 atoms cap the faces of the [Au(La1)_6/6_] prisms, completing the 8-fold coordination of Au, with *d*_Au–La2_ = 3.3208(3) Å. The observed La–Au distances in the La_3_Au_2_ structure are considerably longer than those in La_7_Au_3_, but fall in the range of bonding contacts determined for other compounds.^50^

The breakdown of the La_3_Au_2_ structure into cubic and trigonal-prismatic building blocks allows its description as an intergrowth of W- and AlB_2_-type fragments, as was previously noted for other representatives of this prolific structural family, c.f. Figure 4.^72^

Whereas several Au-containing ternary derivatives of the U_3_Si_2_ type are known, such as Gd_2_Au_2_Sn,^73^ and Ca_2_Au_2_Pb,^74^ the only binary auride reported to crystallize in this type is Y_3_Au_2_.^75^ The latter compound was prepared by annealing the elements at a rather high temperature of 1323 K. A possible explanation for the lack of experimental data for other RE_3_Au_2_ compositions may be their apparently low thermal stability. It is worth noting that an unidentified phase tentatively assigned to the U_3_Si_2_ type was observed upon recent re-evaluation of the La–Mg–Au phase system.^45^ Judging from the estimated lattice parameters (*a* ≈ 8.3 Å, *c* ≈ 4.0 Å), the reported compound may actually be the binary La_3_Au_2_ phase presented here.

#### La_3_Au_4_

**Figure 5:**
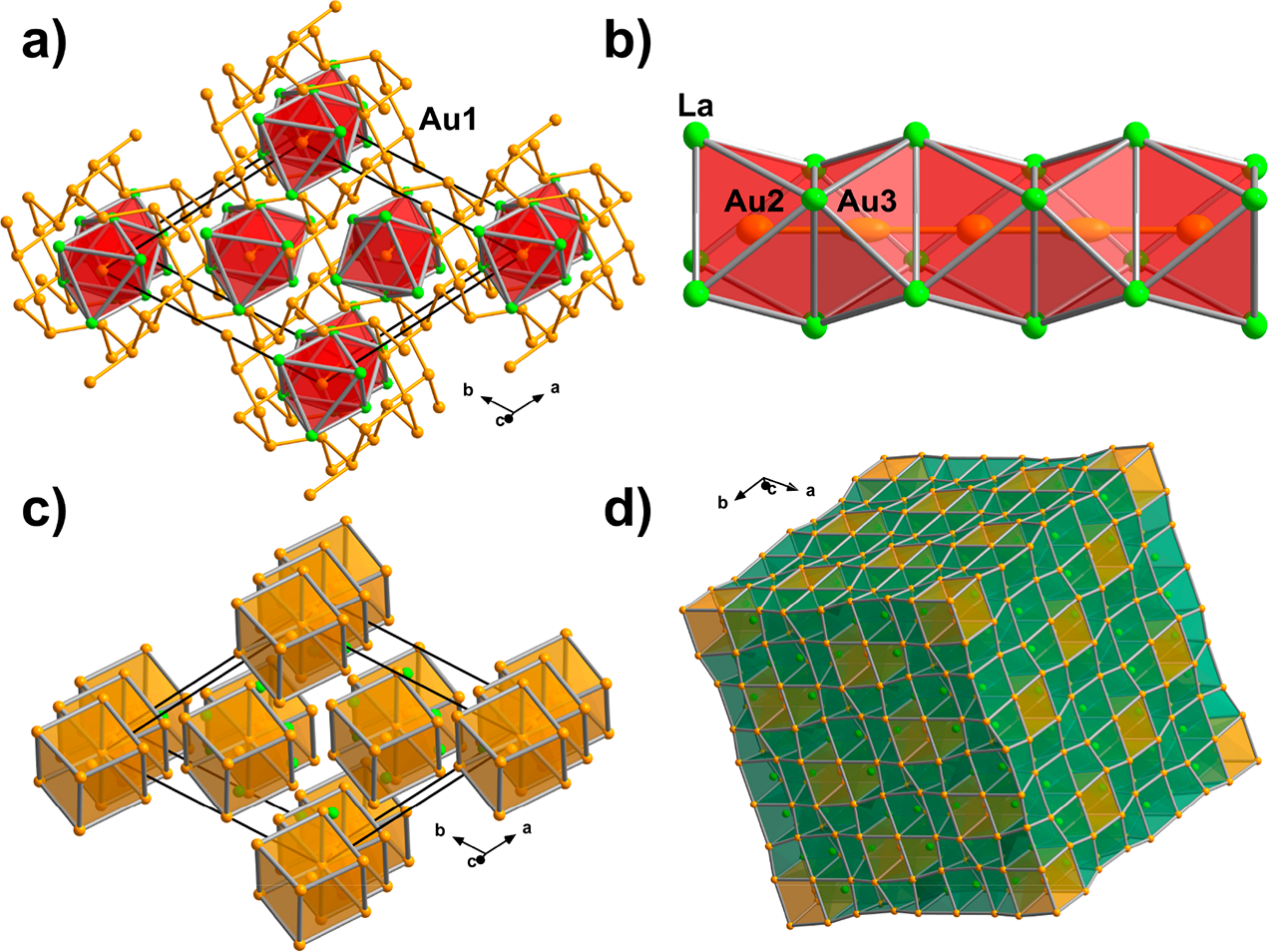
Crystal structure of La_3_Au_4_. (a) Columns of face-sharing [(Au2,3)La_6/2_] octahedra embedded in the three-dimensional Au1 framework. The unit cell (hexagonal setting) is outlined in black. (b) Close-up view of a single column with thermal ellipsoids drawn at the 90% probability level. (c) Corner-sharing Au-centered [(Au2)(Au3)_2/2_(Au1)_6_] distorted cubes and La atoms. (d) Representation as a superstructure of the W type. La- and Au-centered distorted cubes are shown in green and orange, respectively.

In contrast to the previously described phases, La_3_Au_4_ is a new phase identified in the gold-rich field of the phase diagram. It crystallizes in the Pu_3_Pd_4_ type (space group *R*3̅, Pearson code *hR*14, Figure 5). The three crystallographically unique Au sites are distributed between two kinds of polyanionic substructures: Au1 atoms make up a three-dimensional framework hosting large channels running along the *c* direction (hexagonal setting). The Au1–Au1 distances range from 3.0407(8) to 3.1836(6) Å. The channels in turn accommodate linear Au2–Au3– chains with *d*_Au2–Au3_ = 3.1124(8) Å. Both Au2 and Au3 sites are octahedrally coordinated by La, and the resulting octahedra share faces, forming infinite columns  (Figure 5a). Short La–La contacts of 3.654(1) Å are observed between the neighboring columns. Interestingly, the equivalent isotropic displacement parameters for Au2 and Au3 were found to be about 1.7–1.9 times larger than those for Au1 and La. In particular, for Au3, this is reflected in an elongation of the thermal ellipsoid along the *c* direction (Figure 5b). Similar behavior for the corresponding Au sites was reported in isostructural compounds M_3_Au_4_ (M = Ca, Y, Nd).^76−78^ This effect may be related to possible destabilization of the one-dimensional Au chains due to a Peierls distortion. To check for potential pairwise chain breaking, structure refinement was tried in polar space group *R*3. Although it did result in an alternation of the Au–Au distances along the chains with respective values of 3.06(1) Å and 3.16(1) Å, the displacement parameters of Au remained virtually unaffected. Partial breaking of the chains with statistically disordered oligomers or undetected modulation may explain the observed deviations of the displacement parameters. Refinement of the Au sites as split did not yield better results in either *R*3̅ or *R*3, and no obverse–reverse twinning was identified. We note that the analysis of the reciprocal space did not indicate any pronounced diffuse scattering or presence of extra reflections (Figure S2).

Furthermore, single-crystal X-ray diffraction studies at 100 K did not indicate any pronounced distortion either (Tables S1–S3), although the elongation of the thermal ellipsoids was still observed. Therefore, we retained the original structural model in the disorder-free Pu_3_Pd_4_ type.

Although there is no direct Au–Au bonding between the Au1 framework and the (Au2, Au3) linear chains, for a better visualization of the crystal structure, it is convenient to consider the Au2-centered (Au2)(Au3)_2_(Au1)_6_ cluster as a building block. These clusters display a strongly distorted cubic shape due to the long Au2–Au1 distance of 3.7855(4) Å. The “cubes” interconnect by corner sharing, resulting in chains propagating along the *c* direction (Figure 5c). The La atoms occupy the distorted cubic voids between the chains. In this representation, the structure of La_3_Au_4_ can be regarded as consisting of fused AuLa_8_ and LaAu_8_ distorted cubes (Figure 5d), prompting its description as a complex superstructure of the W type. A detailed description of the structural relationship between the Pu_3_Pd_4_ and W types within the Bärnighausen tree formalism^79^ is given in Figure S3.

Numerous compounds containing group 10 elements were reported to crystallize in the Pu_3_Pd_4_ structure type.^80−85^ Among the aurides, structurally well-characterized representatives of this family are limited to M_3_Au_4_ with M = Ca, Y, and Nd.^76−78,86^ In addition, compositions with M = Ce, Pr, Gd, Sm, Tb, and Th were assigned to this structure based on the respective powder X-ray diffraction patterns without further refinements.^78,87−91^ The M_3_Au_4_ phases with rare earth metals Pr, Nd, Gd, and Tb were found to decompose peritectically at 1523–1613 K.^86,88−90^ In light of our experimental data, it is conceivable that La_3_Au_4_ has a similar decomposition point.

#### La_2_Au and α-LaAu

The structures of La_2_Au and α-LaAu were previously assigned to the Co_2_Si (space group *Pnma*, Pearson code *oP*12) and FeB (space group *Pnma*, Pearson code *oP*8) types, respectively, based on powder X-ray diffraction patterns.^92^ In this section, we provide accurate crystal structure determination for the two compounds from single-crystal X-ray diffraction data.

**Figure 6:**
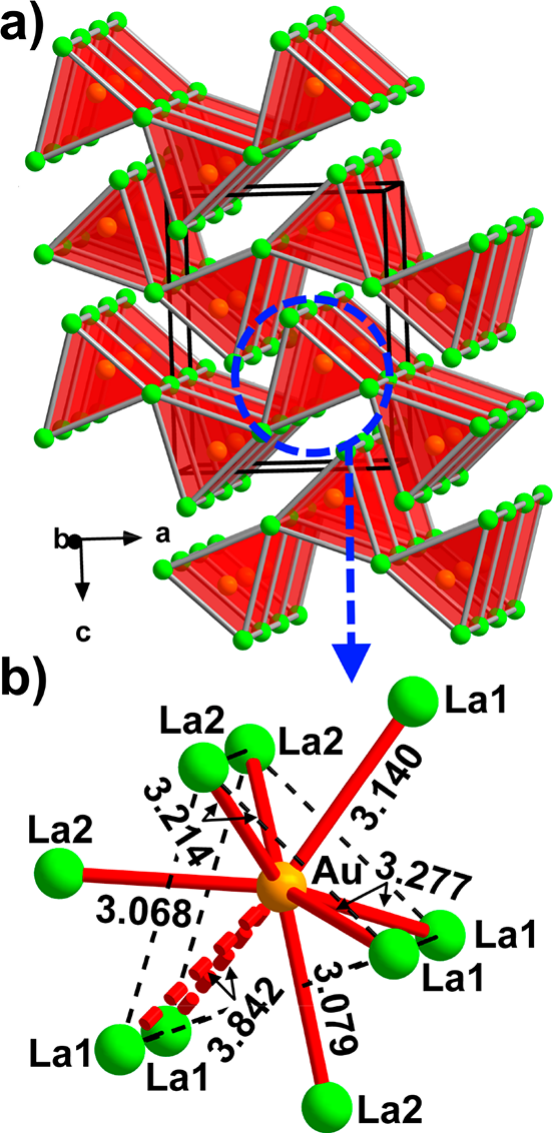
Crystal structure of La_2_Au. (a) Layers of interlinked Au-centered [Au(La2)_2/2_(La1)_4/4_] trigonal prisms. The unit cell is outlined in black. (b) Local coordination environment of the Au site. The distances are given in angstroms. The Au–La contacts that exceed the typical bonding distances are dashed.

The La-richer phase, La_2_Au, can be described as crystallizing in the *anti*-PbCl_2_ structure type (space group *Pnma*, Pearson code *oP*12, Figure 6), which is isopointal to Co_2_Si but displays somewhat different coordination environments due to distinct geometric parameters. McMasters et al. discussed the differentiation between the two structure types in terms of chemical bonding types and argued that the intermetallic Co_2_Si compound bears more similarity to RE_2_Au when electronic interactions are concerned.^92^ In contrast, Chai and Corbett assigned the isostructural Y_2_Au to the *anti*-PbCl_2_ type referring to the similarity in the local atomic coordination.^75^ We prefer to adhere to the latter criterion since the equilibrium geometry of the atomic arrangement, unlike chemical bonding, is an easily measurable quantity. The structures of La_2_Au can be conveniently represented as based on Au-centered AuLa_6_ trigonal prisms which link by sharing the trigonal faces along the *b* direction and by edge sharing along the *a* direction, building up corrugated layers stacked along the *c* axis (Figure 6a). The Au–La distances in the prisms span from 3.068(2) to 3.842(1) Å. The latter value clearly exceeds the typical Au–La bonding distances and demonstrates only weak bonding character according to our calculations (*vide infra*). Disregarding these long Au–La contacts, the Au site is 7-fold coordinated by La, with three of the La atoms coming from the adjacent trigonal prisms (Figure 6b).

**Figure 7:**
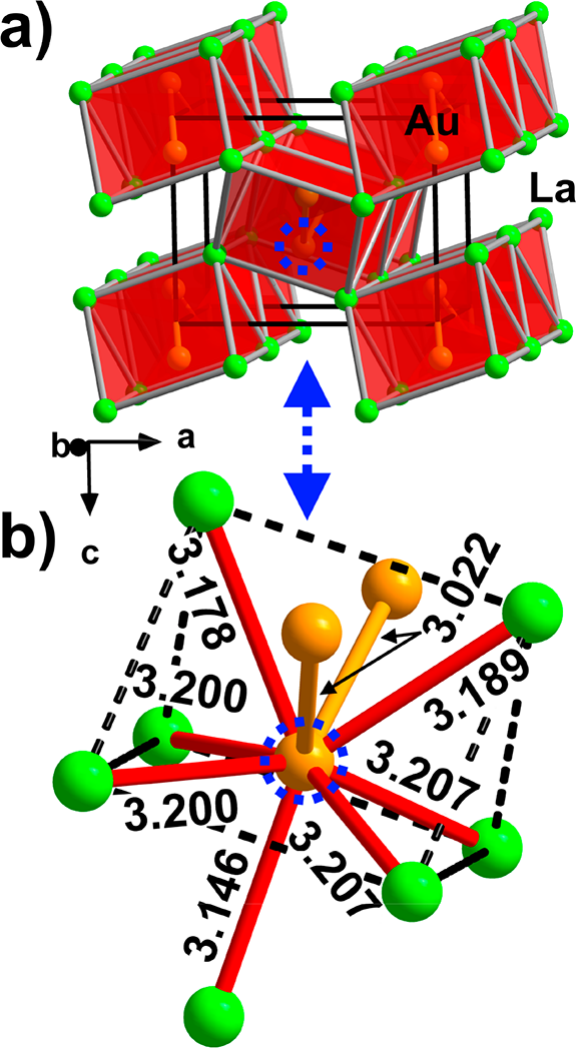
Crystal structure of α-LaAu. (a) Three-dimensional framework emerging from columns of face-sharing Au-centered [AuLa_6/6_] trigonal prisms. The unit cell is outlined in black. (b) Local coordination environment of the Au site. The distances are given in angstroms.

The assignment of the α-LaAu structure to the FeB type (space group *Pnma*, Pearson code *oP*8, Figure 7) is confirmed in our study. Similarly to La_2_Au, the structure can be broken down into Au-centered AuLa_6_ trigonal prisms with *d*_Au–La_ = 3.178(1)–3.189(1) Å. The prisms connect via common rectangular faces giving rise to columns propagating along the *b* axis. The columns interlink by edge and corner sharing to form a three-dimensional framework (Figure 7a). The 7-fold coordination of Au by La is completed by including a La atom from a neighboring prism at a distance of 3.146(1) Å (Figure 7b). In contrast to La_2_Au, where no direct Au–Au interactions are observed, there are infinite zigzag Au chains with *d*_Au–Au_ = 3.0218(7) Å in α-LaAu, running inside the columns along [010].

### Magnetic Properties

**Figure 8:**
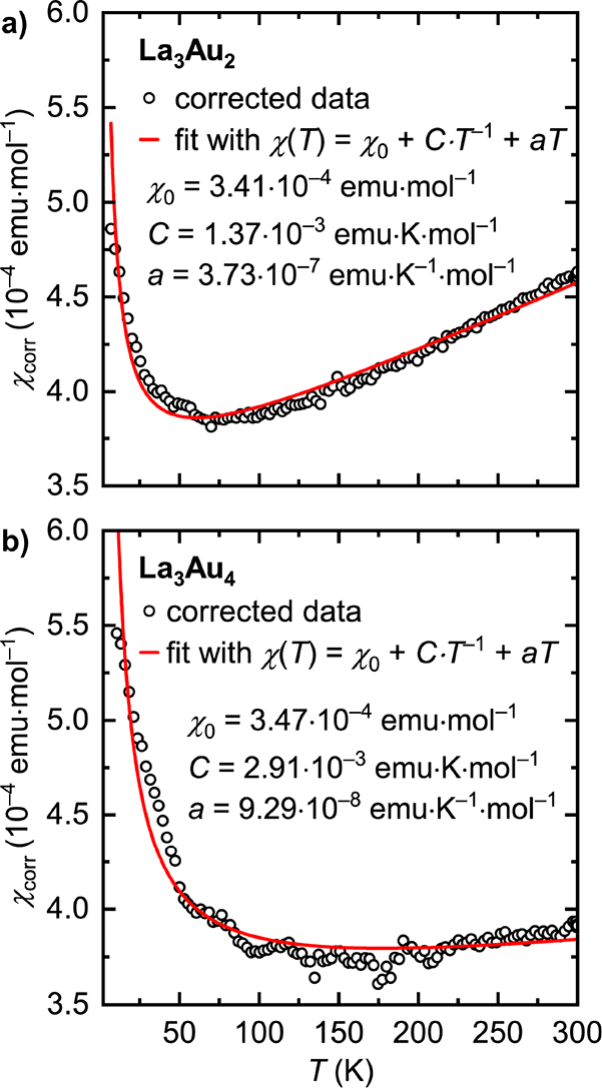
Temperature dependence of the corrected magnetic susceptibility for (a) La_3_Au_2_ and (b) La_3_Au_4_. Open circles and red lines denote the experimental data and nonlinear fits, respectively.

Magnetization measurements were performed for La_3_Au_2_ and La_3_Au_4_. The temperature dependence of the magnetic susceptibility corrected for the sample holder contribution and ferromagnetic impurities is shown in Figure 8. Because of the absence of localized magnetic moments, the magnetic response of La_3_Au_2_ and La_3_Au_4_ is weak. At low temperatures, the magnetic data are affected by paramagnetic impurities, while at high temperatures a linear increase in the magnetic susceptibility with temperature is observed. The latter effect, which is especially pronounced for La_3_Au_2_, is likely associated with the presence of peaks in the electronic density of states around the Fermi level (*vide infra*). To take the paramagnetic impurity and the linear behavior into account, the magnetic susceptibility was fitted with the modified Curie expression, χ(*T*) = χ_0_ + *CT*^–1^ + *aT*, which yielded the temperature-independent contribution χ_0_ of about 3.5 × 10^–4^ emu mol^–1^ for both compounds, indicating the prevalence of the Pauli paramagnetism over the diamagnetic components. Measurements under fields of as low as 10 Oe did not indicate any superconductivity down to 3 K.

### Thermodynamic Stability from First Principles

**Figure 9:**
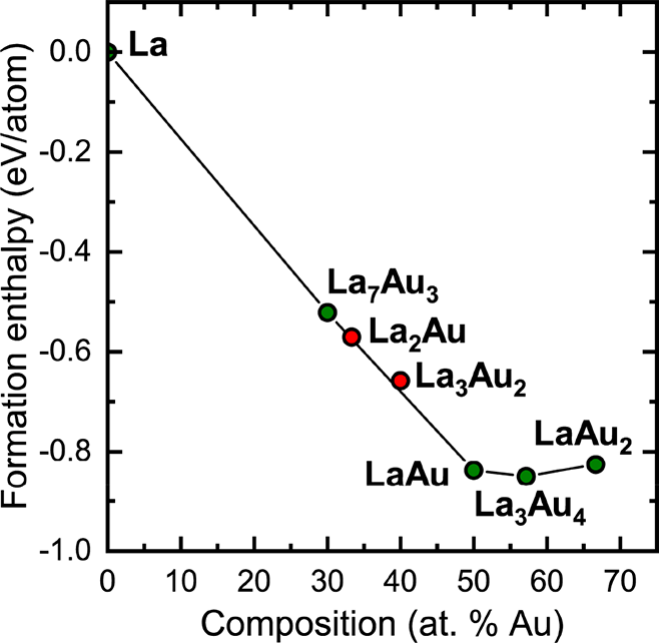
Calculated convex hull for selected binary La–Au phases. Structures located on the hull and above are highlighted in green and red, respectively.

Total energy calculations performed with VASP for the titular phases, selected La–Au binary compounds, and elemental La and Au were used to evaluate formation energies at 0 K and construct an energy convex hull (Figure 9). From this analysis, among the La-rich phases with a La:Au ratio of up to 1:1, LaAu appears to have the highest negative enthalpy of formation per atom, which is also in line with its reportedly high thermal stability. We note here that the two modifications of LaAu, α-LaAu (FeB type) and β-LaAu (CrB type), were found to be almost degenerate in energy. Interestingly, La-richer compositions are located close to the line connecting La and LaAu, which suggests that their formation enthalpies from La and LaAu have very small absolute values. Thus, La_7_Au_3_ is located on the convex hull and is therefore predicted to be thermodynamically stable. However, its decomposition enthalpy into La and LaAu measures only 26.6 meV/atom. Various studies on the reaction enthalpies calculated with DFT methods indicate that the errors in such calculations lie between about 20 and 100 meV/atom for different classes of materials.^93,94^ With this in mind, it can be concluded that the stability of La_7_Au_3_ at 0 K cannot be unambiguously confirmed with DFT methods.

The other two La-rich phases, La_2_Au and La_3_Au_2_, appear to be located above the hull. In this case again, the absolute value of the decomposition enthalpy into La and LaAu is found to be below the expected error of the calculation: 12.1 meV/atom for La_2_Au and 19.8 meV/atom for La_3_Au_2_. The Au-richer composition La_3_Au_4_, which is predicted to be stable, also displays a moderate decomposition enthalpy into LaAu and LaAu_2_ of 17.7 meV/atom. The main conclusion of these computational results is that many binary phases in the La–Au system, including those experimentally confirmed to be thermodynamically stable (such as La_2_Au), exhibit very small decomposition enthalpies, which may result in narrow regions of thermal stability or even metastability at finite temperatures. Since the entropy factor is not taken into account in such calculations, in general, an analysis of thermodynamic stability at 0 K should always be taken with care, especially when the enthalpy differences are small.

We conclude our discussion of the DFT-derived thermodynamics with a note on the possible stabilization of the La_7_Au_3_ structure with a foreign element. Since the mentioned compound could not be prepared single-phase, a natural question to ask is whether the presence of some undetected third element is responsible for the stabilization of this phase. Our SCXRD analysis allows ruling out elements of the second period (such as carbon, nitrogen, and oxygen) and heavier elements (such as Mo from the reactor) as possible constituents of the crystal structure, since any significant amounts of these elements would be detectable. On the basis of the analysis of the X-ray diffraction data, we cannot exclude the presence of some hydrogen, which was found to be responsible for the stabilization of many seemingly binary phases in the past.^28^ However, our attempt to prepare La_7_Au_3_ under hydrogenating conditions resulted in the formation of LaH_2_ and did not produce the target phase (*vide supra*). It is not clear if the employed reaction conditions were too harsh, e.g., if the hydrogen pressure was too high. For this reason, we also investigated the stability of the La_7_Au_3_ structure upon incorporation of hydrogen from first principles.

As discussed above, the crystal structure of La_7_Au_3_ features some tetrahedral and octahedral voids surrounded by La atoms. These voids could potentially accommodate H atoms. To check this hypothesis using first-principle calculations, we considered three model structures: La_7_Au_3_H(tetr), with H atoms placed exclusively in the tetrahedral voids; La_7_Au_3_H(oct), with H atoms in the octahedral voids only; and La_7_Au_3_H_2_, with H atoms occupying both kinds of voids. The structures were fully relaxed, and their stability was checked with respect to other phases in the La–Au–H system. We found that in all cases the introduction of H into the structure has a destabilizing effect and the formation of the binary hydride LaH_2_ is favorable:
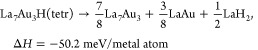

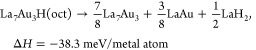

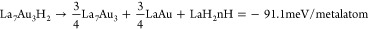
Two points need to be mentioned here. First, the nature of the Au-containing product in our reaction under hydrogen is not yet known, so it cannot be considered for the calculations. Second, the given absolute values of the decomposition enthalpies are not particularly high and may be regarded as lying on the upper side of the expected calculation error. Nevertheless, the observed trend suggests that placing hydrogen in the tetrahedral or octahedral voids in the structure of La_7_Au_3_ will not have a stabilizing effect. Of course, other factors such as the potential location of hydrogen in other parts of the structure or entropy stabilization of a hydride should also be considered. In conclusion, although the presence of hydrogen in the experimentally observed La_7_Au_3_ cannot be completely ruled out, our experimental data and first-principles calculations do not support this scenario.

### Thermal Analysis

**Figure 10:**
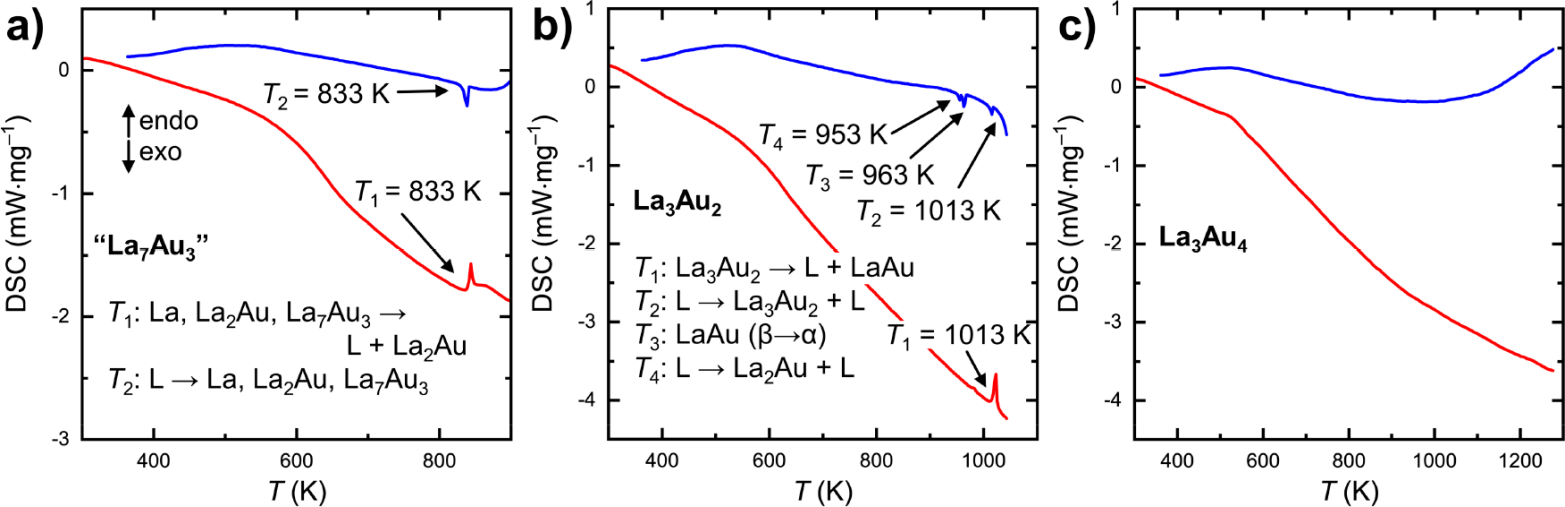
Differential scanning calorimetry on heating (red) and cooling (blue) for La_7_Au_3_ (a, about 30 wt % La_7_Au_3_ in the sample), La_3_Au_2_ (b), and La_3_Au_4_ (c). Thermal events with the corresponding temperatures and explanations are indicated. See the text for details.

Results of the differential scanning calorimetry (DSC) analysis for a sample containing about 30 wt % La_7_Au_3_ are shown in Figure 10a. Upon heating, the sample undergoes incongruent melting at about 833 K, corresponding to the eutectic point between elemental La and La_2_Au.^44^ No other effects are seen below this temperature, indicating that the decomposition of La_7_Au_3_ must be too slow to occur within the time frame of the measurement. Upon cooling, the sample crystallizes with virtually no supercooling effect. No other transitions are visible below the solidification point. PXRD analysis of the sample after the DSC measurement revealed the presence of La, La_2_Au, and small amounts of La_7_Au_3_ (Figure S4). From the described behavior, it can be inferred that La_7_Au_3_ is a metastable phase, which forms upon crystallization from the melt in line with Ostwald’s rule and decomposes upon melting or prolonged heating at a temperature sufficient for solid-state diffusion to occur.

DSC measurements on a La_3_Au_2_ sample (Figure 10b) revealed an endothermic peak upon heating to 1013 K. No other intrinsic transitions are observed below this temperature; a small bump in the heating curve at about 963 K corresponds to the polymorphic transition (α → β) of a tiny amount of LaAu impurity in the sample. Ex situ analysis of a La_3_Au_2_ sample annealed above 1013 K suggests that a peritectic decomposition into LaAu and a La-rich melt occurs at this temperature. From the DSC analysis, the enthalpy of melting for La_3_Au_2_ was estimated to be 16.8 kJ/mol (or 34.8 meV/atom, i.e, within the error range for reaction enthalpy estimation using DFT methods). Upon cooling, the DSC curve reveals three exothermic effects: the crystallization of La_3_Au_2_ (*T* = 1013 K), the polymorphic β → α transition of LaAu (*T* = 963 K), and the crystallization of La_2_Au from the melt (*T* = 953 K).

Finally, our DSC analysis of a La_3_Au_4_ sample at temperatures of up to 1273 K did not reveal any thermal effects (Figure 10c), suggesting that the decomposition or melting occurs at higher temperatures.

**Figure 11:**
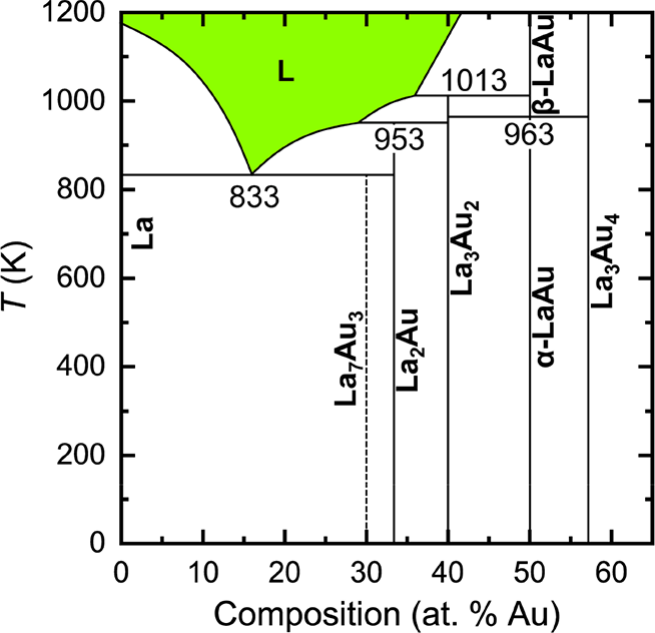
Part of the re-evaluated La–Au phase diagram. Polymorphic phase transitions in elemental La are omitted for clarity. The metastable La_7_Au_3_ is depicted with a dashed line. Selected temperatures (K) are indicated.

With the collected data at hand, we are able to propose a refined version of the La–Au binary phase diagram in the La-rich region (Figure 11). While the phase relationships involving La_3_Au_4_ remain unclear, important updates to the published phase diagram include the incongruently melting La_3_Au_2_ and the metastable La_7_Au_3_.

### Electronic Structure and Chemical Bonding

**Figure 12:**
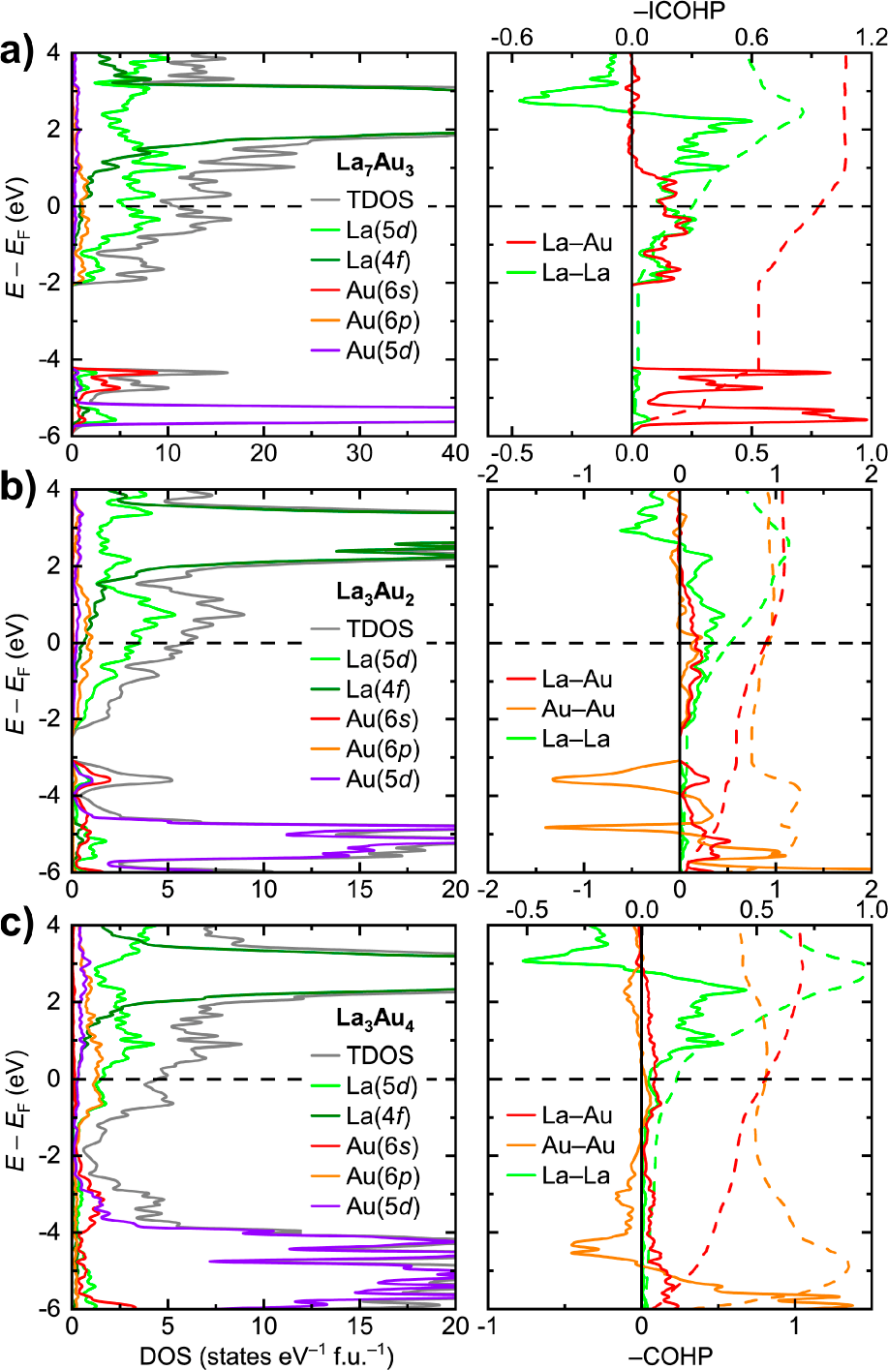
Total and projected electronic densities of states (DOS) and bond-averaged crystal orbital Hamilton population curves (COHP) for (a) La_7_Au_3_, (b) La_3_Au_2_, and (c) La_3_Au_4_. Dashed lines denote integrated COHP curves.

A first attempt to rationalize the electronic structure and bonding of La_7_Au_3_, La_3_Au_2_, La_3_Au_4_, La_2_Au, and α-LaAu by applying the Zintl–Klemm formalism (which holds true for many polar intermetallics) allows rewriting the compounds’ formulas as (La^3+^)_7_(Au^–^)_3_(e^–^)_18_, (La^3+^)_3_(Au^–^)_2_(e^–^)_7_, (La^3+^)_3_(Au^–^)_4_(e^–^)_5_, (La^3+^)_2_(Au^–^)(e^–^)_5_, and (La^3+^)(Au^–^)(e^–^)_2_, respectively, if occupation of the Au(6p) states is not taken into account for the calculation of the formal charge. Thus, they would be expected to be electron-rich, polar intermetallics. In line with this simplified evaluation, the electronic densities of states (DOS) for La_7_Au_3_, La_3_Au_2_, La_3_Au_4_, La_2_Au, and α-LaAu (Figure 12a–c and Figure S5a,b) reveal metallic character and sizable charge transfer. At the Fermi level (*E*_F_), the DOS are dominated by the La(5d) states with a small contribution of Au(6p) and Au(5d). Unoccupied La(4f) states form a peak in the DOS centered at about *E* – *E*_F_ = 2 to 3 eV. The Au(6s) and Au(5d) components are mostly localized well below *E*_F_, confirming the anionic nature of Au. With the emergence of Au–Au bonding, the dispersion of the Au(6s) and Au(5d) states gradually increases. Thus, in La_7_Au_3_ (Figure 12a) and La_2_Au (Figure S5a), both lacking direct Au–Au interactions, a domain with Au(6s, 5d) character is situated below *E* – *E*_F_ = −4 eV and is separated from the bands crossing the Fermi level by an energy gap. In La_3_Au_2_ (Figure 12b) and α-LaAu (Figure S5b), with isolated Au dumbbells and zigzag Au chains, respectively, the Au(6s) and Au(5d) states broaden and the gap between these states and the higher-lying bands is reduced. Finally, in La_3_Au_4_ (Figure 12c), with an extended three-dimensional framework of Au–Au bonds, the Au(6s) and Au(5d) regions become continuous in a wide energy window, with a dip in the DOS at around *E* – *E*_F_ = −2 eV. Within the framework of the rigid band approximation, the location of the gap or the dip in the electronic spectra of La_7_Au_3_, La_3_Au_2_, La_3_Au_4_, La_2_Au, and α-LaAu corresponds to the removal of 18, 7, 5, 5, and 2 electrons per formula unit, respectively. These numbers are in perfect agreement with the electron excess calculated above. This shows that the electronic structure of the studied materials can be fairly well explained using, as the first approximation, simple electron counting suitable for compounds with highly localized bonding and augmenting this picture with electronic delocalization, an approach that appears to be applicable for a great number of polar intermetallic phases.^95^

Crystal orbital Hamilton population analysis (COHP) revealed that for all studied compounds, the La–Au contacts show exclusively bonding character below *E*_F_, with some extra bonding states available just above the Fermi level, resulting in slightly underoptimized interactions (Figure 12a–c). The long La–Au contacts in the range of 3.65–3.84 Å, observed in La_7_Au_3_ and La_2_Au, were also examined, but were found to exhibit considerably smaller bonding contributions in comparison with the shorter La–Au pairs.

The Au–Au bonding, observed for La_3_Au_2_, La_3_Au_4_, and α-LaAu, displays a more complex electronic pattern hallmarked by a combination of bonding and antibonding states below *E*_F_. Nevertheless, the magnitude of the bond-averaged negative integrated COHP values (−ICOHP) for the Au–Au contacts at the Fermi level is comparable to that of the La–Au bonds. Interestingly, for La_3_Au_4_, all Au–Au contacts, except Au2–Au3 along the Au chains, demonstrate bonding states in the vicinity of *E*_F_. In contrast, in the case of Au2–Au3, the Fermi level crosses an extended region of antibonding character, spanning from about *E* – *E*_F_ = −2.4 to 1.1 eV (Figure S6). Although, the integration over all states below *E*_F_ indicates a net attractive interaction for Au2–Au3, with an −ICOHP value close to those of other Au–Au contacts, the location of *E*_F_ in the antibonding domain suggests that the Au–Au chains in La_3_Au_4_ are too electron-rich and may be prone to destabilization, such as Peierls distortion, discussed above in relation to the increased atomic displacement parameters of the Au atoms in the chains. To further study the possibility of such a distortion, we attempted geometry optimizations for La_3_Au_4_ with the VASP code starting from the ideal structure with equidistant Au chains and from a hypothetical structure with a pairwise distortion of the chains. Both optimization runs converged to essentially the same structure with identical Au–Au distances along the chain. The introduction of spin–orbit coupling did not affect this result. This observation suggests that structural distortion, if any, happens on scales larger than the size of a single unit cell. In order to spot such a distortion experimentally, crystallographic studies at helium temperatures may be necessary.

Since some of the presented structures exhibit relatively short La–La contacts, this kind of homoatomic interaction was also analyzed. Only La–La pairs with interatomic distances of less than 3.90 Å were considered. In all studied compounds, such contacts are characterized exclusively by bonding states below *E*_F_. However, due to a transfer of electron density from the La atoms to the Au species, the resulting La–La bonding interactions turn out to be too electron-deficient and consequently much weaker than the La–Au bonding, yet non-negligible. In particular, for La_3_Au_2_, the −ICOHP magnitude for the short La–La contact with *d*_La–La_ = 3.75 Å amounts to about 50% of the bond-averaged La–Au value. This bonding picture is reminiscent of that in the lanthanum subiodide LaI, which also exhibits unsaturated, but still quite pronounced, La–La bonding.^96^

## Conclusions

In this contribution, we described three new binary phases in the La–Au system—La_7_Au_3_, La_3_Au_2_, and La_3_Au_4_—crystallizing in known structure types, which are nevertheless rather uncommon for binary gold-containing intermetallic compounds. In addition, we determined the crystal structures of known binaries La_2_Au and α-LaAu. An examination of the crystal and electronic structures of the listed materials suggests that the increase in the Au content is accompanied by the emergence of structural units with homoatomic Au–Au bonding, such as Au_2_ dimers in La_3_Au_2_, Au chains in LaAu, and extended Au frameworks in La_3_Au_4_.

Our exploratory studies, first principles calculations, and thermal analysis indicate that the La-richest phase La_7_Au_3_ is metastable and can be prepared by crystallization from a stoichiometric melt, in accordance with Ostwald’s rule. In contrast, La_3_Au_2_ and La_3_Au_4_ appear to be thermodynamically stable. The former phase decomposes peritectically at 1013 K, while the latter is stable at least up to 1273 K. Our investigations enabled a re-evaluation of the available La–Au phase diagram in the La-rich part. Taking into account the relative scarcity of detailed thermodynamic studies devoted to gold-containing intermetallics, it is not surprising that even binary systems may offer a source of new compounds with potentially interesting crystal-structural characteristics and physical properties. In this respect, the observed propensity of Au in the formation of homoatomic bonds makes it interesting to explore Au-rich phase spaces as well.

## Supporting Information Available

Crystal structure data for La_3_Au_4_ at *T* = 100 K, reciprocal space reconstruction for La_3_Au_4_, additional PXRD data, group–subgroup relationships for Pu_3_Pd_4_ and *bcc* structures, and supplementary electronic structure and chemical bonding analysis (PDF)

## Accession Codes

CCDC 2072067–2072071 contain the supplementary crystallographic data for this paper. These data can be obtained free of charge via www.ccdc.cam.ac.uk/data_request/cif, or by emailing data_request@ccdc.cam.ac.uk, or by contacting The Cambridge Crystallographic Data Centre, 12 Union Road, Cambridge CB2 1EZ, UK; fax: +44 1223 336033.
