# Supplementary material for: Overlooked Binary Compounds Uncovered in the Reinspection of the La–Au System: Synthesis, Crystal Structures, and Electronic Properties of La7Au3, La3Au2, and La3Au4
Source: Inorg Chem. 2021 Jul 28;60(16):12158–71. doi: 10.1021/acs.inorgchem.1c01355 (PMC8389835; doi:10.1021/acs.inorgchem.1c01355)
Supplement: Supplementary file 1 — ic1c01355_si_001.pdf [file ic1c01355_si_001.pdf]

Supporting Information

Overlooked binary compounds uncovered in the re-inspection of the La–Au system: Synthesis, crystal structures, and electronic properties of La_7Au_3, La_3Au_2, and La_3Au_4

Alexander Ovchinnikov and Anja-Verena Mudring*

Department of Materials and Environmental Chemistry, Stockholm University, Svante Arrhenius väg 16 C, 10691 Stockholm, Sweden.

E-mail: anja-verena.mudring@mmk.su.se

Contents: 3 Tables, 6 Figures

Table S1. Refinement details and selected crystallographic data for La_3Au_4 (T = 100 K, Mo Kα λ = 0.71073 Å)

Refined composition	La_3Au_4
fw/ g mol^{-1}	1204.60
Space group	$R\bar{3}$ (# 148)
Z	6
a/ Å	14.035(2)
c/ Å	6.1910(9)
V/ Å^3	1056.1(4)
ρ_{calc}/ g cm^{-3}	11.364
$\mu_{MoK\alpha}$/ mm^{-1}	100.67
R_{int}	0.076
R_1 [$I > 2\sigma(I)$]^a	0.036
wR_2 [$I > 2\sigma(I)$]^a	0.084
R_1 [all data]^a	0.038
wR_2 [all data]^a	0.085
$\Delta\rho_{max,min}$/ e Å^{-3}	2.85, –3.52

$^a R_1 = \sum||F_o| - |F_c||/\sum|F_o|$; $wR_2 = [\sum[w(F_o^2 - F_c^2)^2]/ \sum[w(F_o^2)^2]]^{1/2}$, where $w = 1/[\sigma^2 F_o^2 + (0.0311P)^2 + (147.4296P)]$ and $P = (F_o^2 + 2F_c^2)/3$.

Table S2. Atomic coordinates and equivalent isotropic displacement parameters (Å^2) for La_3Au_4 (T = 100 K)

Atom	Site	x	y	z	U_{eq}^{a}
La	18f	0.04400(8)	0.21236(9)	0.23200(16)	0.0221(3)
Au1	18f	0.39028(5)	0.11437(5)	0.04952(11)	0.0192(3)
Au2	3b	0	0	1/2	0.0331(5)
Au3	3a	0	0	0	0.0447(6)

$^a U_{eq}$ is defined as one third of the trace of the orthogonalized U_{ij} tensor.

Table S3. Anisotropic displacement parameters (Å^2) for La_3Au_4 (T = 100 K)

Atom	U_{11}	U_{22}	U_{33}	U_{12}	U_{13}	U_{23}
La	0.0187(5)	0.0288(6)	0.0188(5)	0.0120(4)	–0.0004(4)	0.0000(4)
Au1	0.0196(4)	0.0192(4)	0.0195(4)	0.0102(3)	0.0014(2)	0.0009(2)
Au2	0.0234(6)	U_{11}	0.0524(12)	1/2U_{11}	0	0
Au3	0.0190(6)	U_{11}	0.0961(19)	1/2U_{11}	0	0

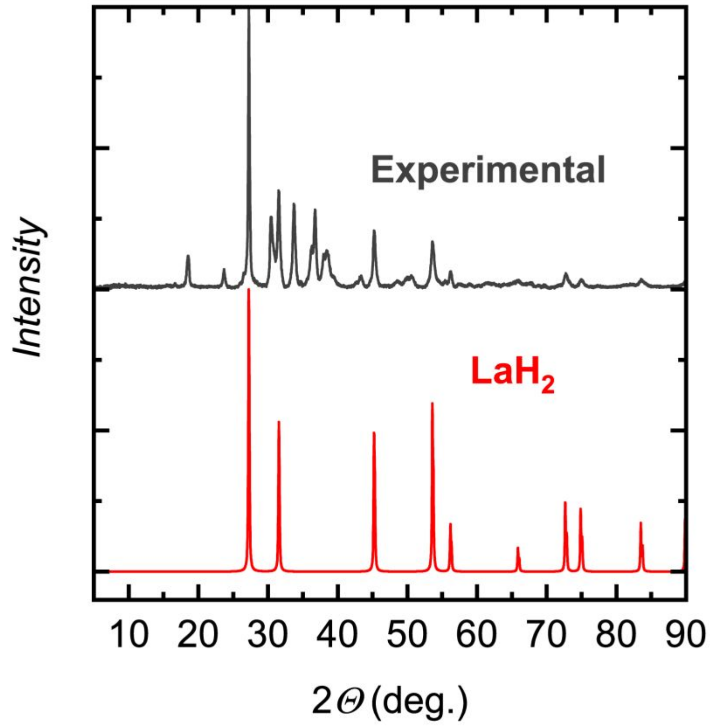

Figure S1. Powder X-ray diffraction pattern (Cu Kα) of a "La_7Au_3" sample after the hydrogenation reaction. Experimental data with subtracted background and theoretical powder pattern for LaH_2 are shown in grey and red, respectively. The unassigned broad peaks belong to an unidentified phase or a mixture of phases.

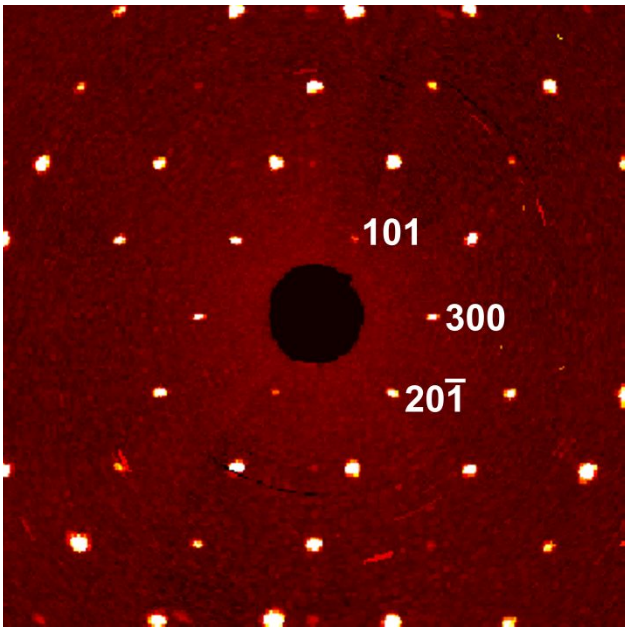

Figure S2. Synthesized precession image of the *h*0*l* layer for La_3Au_4 (room temperature). Selected reflections are labeled.

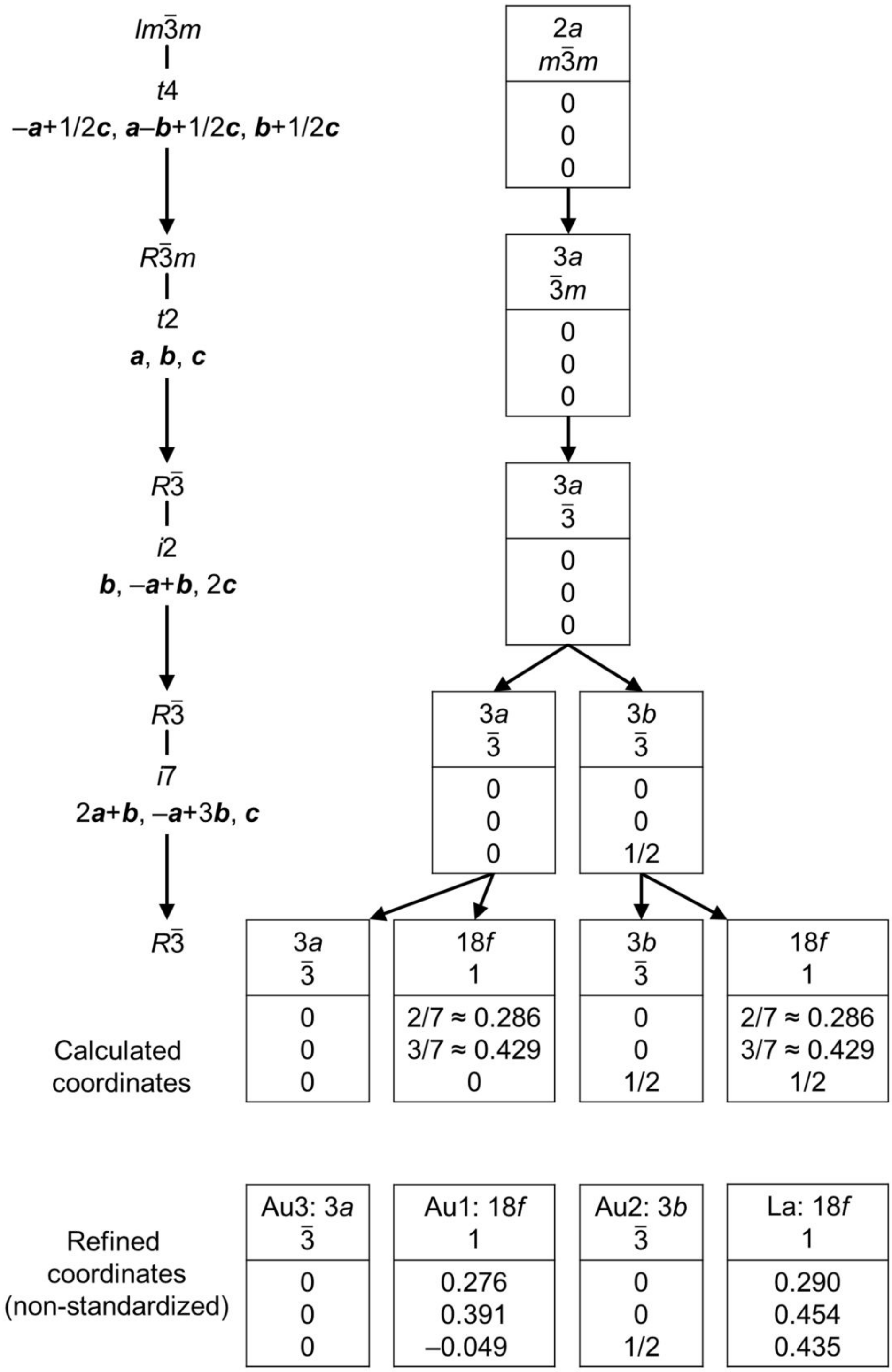

Figure S3. Group-subgroup scheme for the simple *bcc* structure (W type) and La_3Au_4 (Pu_3Pd_4 type). The indices for *translationengleiche* (*t*), isomorphic (*i*) and non-isomorphic (*k*) *klassengleiche* symmetry reductions, unit cell transformations, and evolution of the atomic parameters are shown. The ideal *c*/*a* ratio for the Pu_3Pd_4 type from the unit cell transformations is $\sqrt{3/14} \approx 0.463$. Experimental *c*/*a* ratio for La_3Au_4 is 0.443.

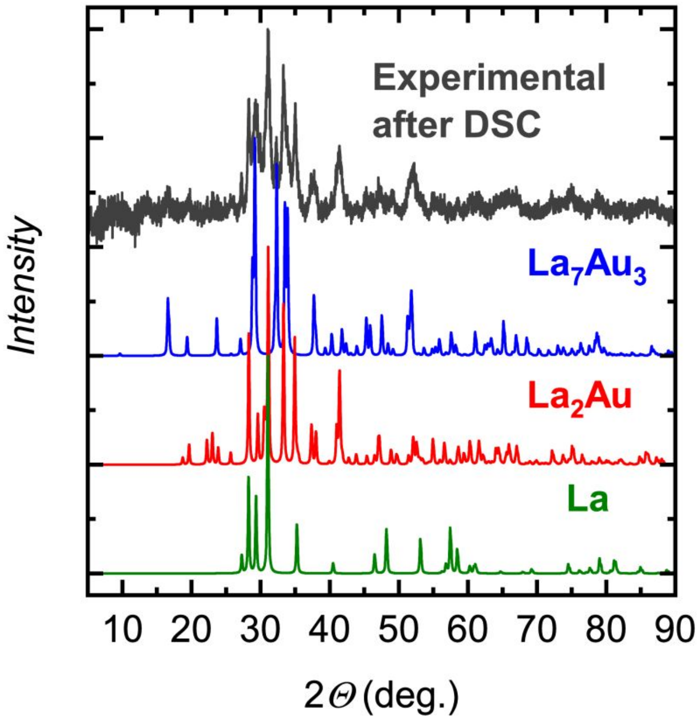

Figure S4. Powder X-ray diffraction pattern (Cu Kα) of a “La_7Au_3” sample after DSC measurement. Experimental data with subtracted background and theoretical powder patterns for La_7Au_3, La_2Au, and La are shown in grey, blue, red, and green, respectively.

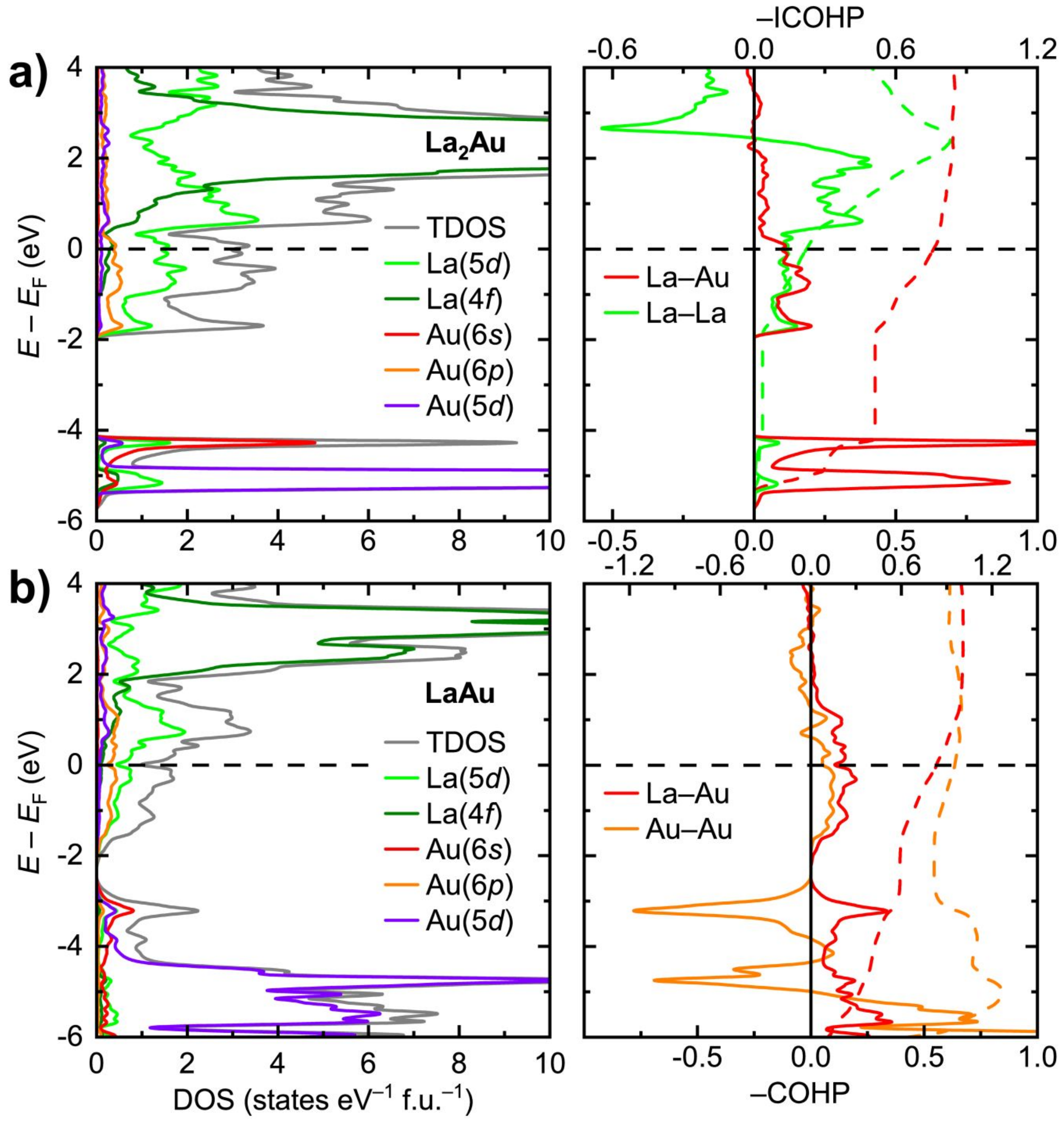

Figure S5. Total and projected electronic densities of states (DOS) and bond-averaged Crystal Orbital Hamilton Population curves (COHP) for La_2Au (a) and α-LaAu (b). Dashed lines denote integrated COHP curves.

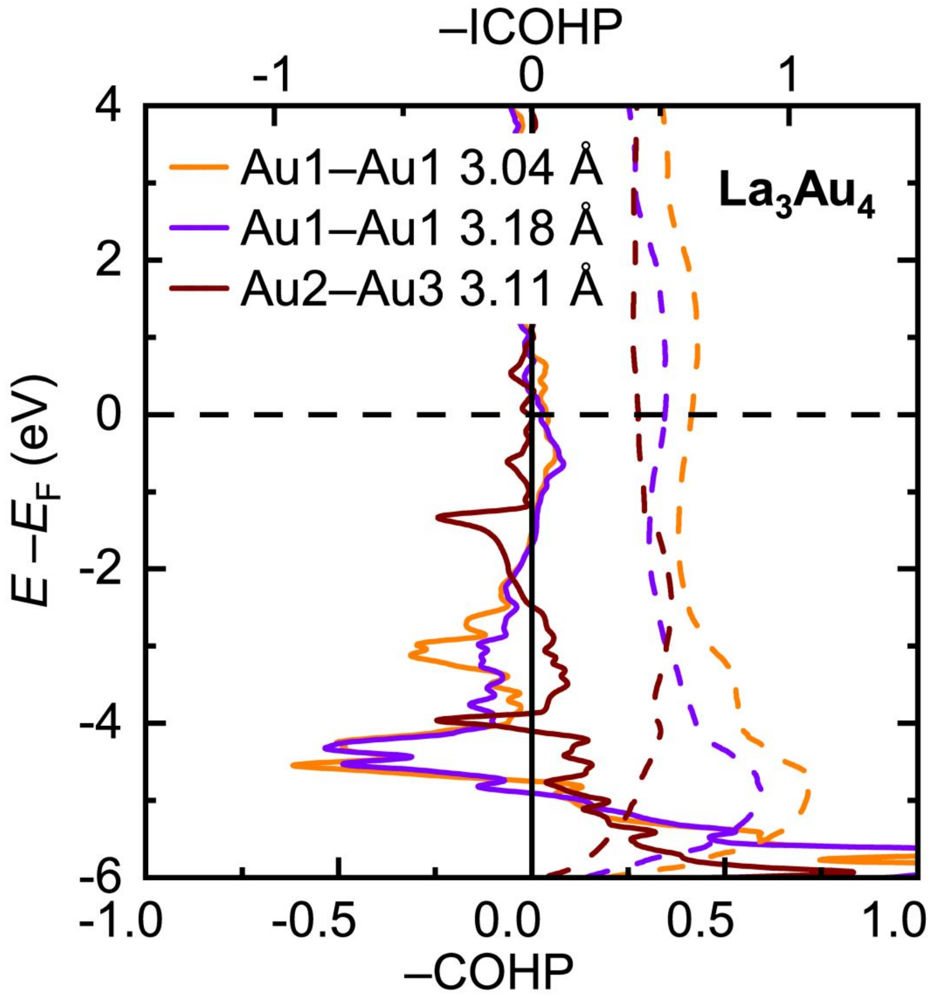

Figure S6. Crystal Orbital Hamilton Population curves (COHP) for individual Au–Au contacts in La_3Au_4. Dashed lines denote integrated COHP curves.
